# Supplementary material for: Industrial technological advance and the employment demand for China’s labour force—Micro evidence from labour hiring in companies
Source: PLoS One. 2024 Nov 19;19(11):e0307564. doi: 10.1371/journal.pone.0307564 (PMC11575794; doi:10.1371/journal.pone.0307564)
Supplement: Attachment — Submitted filename: Response to Reviewers.docx [file pone.0307564.s002.docx]

**Response to academic editor**

1. **Please ensure that your manuscript meets PLOS ONE's style requirements, including those for file naming.**

**Response：**Following your suggestions, the authors have modified the article formatting according to the PLOS ONE style template, renaming the files according to the file naming requirements.

2. **Please include captions for your Supporting Information files at the end of your manuscript, and update any in-text citations to match accordingly.**

**Response：**As per your request, the authors have added the captions of the supporting information files at the end of the manuscript and updated any in-text citations to match accordingly.

**Reviewer #1**

**1. This article needs to further clarify the difference between this article and the existing literature, because there is a lot of literature studying this issue, such as literature studying the impact of artificial intelligence, automation, digital transformation, etc. on labor demand.**

**Response：**Previous literature is mainly about macro-level technological advances as well as micro-level technological advances, while there is an extreme lack of relevant research on the industrial technology level. In practice, industrial technological advances have caused changes in the demand, structure and quality of labour hiring in companies, and some scholars (Dauth et al., 2018) have begun to pay attention to the possible impact of specific technologies such as robotics applications, artificial intelligence, automation, digital transformation and other technologies applied in companies on the labour market in China, but the impact of industrial technologies in a broad sense on the labour hiring decisions of companies and their mechanisms of action has not yet been carried out. This paper provides an in-depth analysis of the evolutionary process of industrial technology, and refines and summarizes the formation, scope and nature of industrial technological advances, based on which it theoretically extrapolates and empirically examines its impact mechanism on company labour hiring, expanding the study of the microeconomic consequences of industrial technology.

Industrial technology suffers from a nonlinear evolutionary process in which technologies evolve from discrete distributions that do not interfere with each other to highly agglomerative network structures that evolve synergistically (Antonio et al., 2010). The fundamental nature of industrial technology is complexity (Hanieh and Arash, 2023), and industrial technology complexity specifically exhibits the characteristics of non-linearity, unpredictability, divisibility and systemicity (Fang et al., 2023). The relevant literature mainly focuses on the fragmentation of ‘theoretical elaboration’, ‘measurement’ (Mewes and Broekel, 2022) and ‘influencing factors’, and has not yet formed a more systematic theoretical study. A more systematic theoretical system has not yet been formed. Previous research on specific technological advances such as artificial intelligence, automation, digital transformation, etc. has not examined the influence mechanism from the perspective of these characteristic facts. In this paper, the analysis of labour employment based on the characteristics of industrial technological advances is obviously more in line with the logic of reality and the paradigm of theoretical deduction, and it is also an enrichment and supplementation of the original industrial technology theory.

**2. This article chooses TFP to measure technological progress, which is certainly possible, but it is recommended that the authors further use other standard methods to measure TFP, such as LP and OP methods, etc., so as to ensure the robustness of the results.**

**Response：**Thanking the experts for their valuable comments, the authors use LP and OP methods to calculate the total factor productivity of companies and take the industry average as a proxy variable for industrial technological advancement, and the results, as shown in columns (3) and (4) of Table 5, show that the regression coefficients of LP and OP are significantly negative at the 5 per cent level, a result which further supports the findings of this paper.

Table 5 Key variable substitution

|  | （3）  *lnNumber* | （4）  *lnNumber* |
| --- | --- | --- |
| *LP* | -0.2220*** |  |
|  | (-24.5799) |  |
| *OP* |  | -0.3188*** |
|  |  | (-44.9943) |
| *_cons* | -5.9925*** | -8.7625*** |
|  | (-13.6626) | (-19.7455) |
| *Control* | Yes | Yes |
| *City* | Yes | Yes |
| *Year* | Yes | Yes |
| N | 40584 | 36431 |
| *p>\|t\|* | p<0.001 | p<0.001 |
| Adj R-sq | 0.6074 | 0.6218 |

3. **The benchmark regression in this article has serious endogeneity problems, and it is recommended that the author select instrumental variables for estimation.**

**Response：**In view of your constructive comments, the authors chose as an instrumental variable the distance (IV) from each province and district to the nearest port.the distance from each province and region to the port reflects the front-end, middle-end and back-end locality of the technological division of labour in the industry chain with differences in the market environment, and different technological positions will depend on differences in the market environment. Therefore, foreign market proximity forms an important external environment for industrial technological progress, can screen local industrial technology with high correlation with the formation of industrial technological progress, but does not directly affect enterprise labour employment, and is not correlated with enterprise labour employment and the residual term of the model, which can be used as an instrumental variable for industrial technological progress. The instrumental variable Cragg-Donald Wald F-statistic is greater than the 15% level critical value, indicating that there is no problem of weak instrumental variables, and the p-value is significant at the 1% level in the non-identifiability test and the endogeneity test, i.e. the original hypothesis is rejected. Column (3) of Table 6 presents the results of the second stage regression, where the coefficients of the key explanatory variables are all significantly negative at the 1 per cent level. This indicates that the results remain robust after controlling for possible endogeneity issues.

Table 6	robustness tests

|  | （2）  *tfp* | （3）  *lnNumber* |
| --- | --- | --- |
|  | *Instrumental variable test* | |
| tfp |  | -6.5982*** |
|  |  | (-7.7893) |
| *IV* | -0.0064*** |  |
|  | (-9.2318) |  |
| *_cons* | 0.8961*** | -3.1376*** |
|  | (38.2367) | (-4.6335) |
| *Control* | Yes | Yes |
| *City* | No | No |
| *Year* | Yes | Yes |
| N | 40601 | 40601 |
| *p>\|t\|* | p<0.001 |  |
| Adj R-sq | 0.4407 |  |

**4. The author selects a policy as an exogenous shock and constructs a difference-in-difference model, but the author does not explain well why this policy can represent technological progress.**

**Response：**According to your suggestion, the authors use the policy that is more representative of technological advance: the establishment of national new zones as an exogenous shock to construct the DID model, combing through the relevant studies on the policy of national new zones, based on which, they further explain the relationship between the policy of national new zones and technological advancement as follows: industrial technological advancement tends to be correlated with the degree of marketisation and openness in the region, and national new zones have a driving effect on regional economic growth and belong to comprehensive economic functional zones, which are more likely to realise industrial agglomeration and technological innovation and advance industrial technology in the region.

**5. This article did not conduct a mechanism test.**

**Response：**Modified to add the mechanism test.

# 6. **Mechanism test**

**6.1 Incentive effects: a test of investment expansion effects and chain transmission effects**

**6.1.1 Technology investment expansion effect**

The previous theoretical analysis suggests that industrial technological advances generate investment expansion effects and increase company labour employment. This paper uses the ratio of company R&D investment to total assets to measure the scale of enterprise investment, table 9, column (1) column (1) of the estimation results of industrial technological advances on the scale of enterprise investment, industrial technological advances in the 1 per cent level is significantly positive, technological investment expansion effect exists.

Table 9 Technology investment expansion effects and chain transmission effects

|  | (1) | (2) | (3) | (4) | (5) |
| --- | --- | --- | --- | --- | --- |
|  | *lnincome* | *lnNumber* | *lnNumber* | *lnNumber* | *lnNumber* |
|  | *Technology investment expansion effect* | *Upstream pass-through effect (industry i is upstream)* | | *Downstream effects (industry i is downstream)* | |
| tfp | 0.0039*** |  | -0.9529*** |  | -0.8041*** |
|  | (4.9987) |  | (-23.7675) |  | (-24.5487) |
| *Upstream_ct_* |  | 0.2553*** | 0.5697*** |  |  |
|  |  | (3.1918) | (7.1246) |  |  |
| *Dowmstream_ct_* |  |  |  | 0.5668*** | 0.7941*** |
|  |  |  |  | (5.6408) | (7.9499) |
|  |  |  |  |  |  |
| *_cons* | 0.0493*** | -5.6118*** | -4.8197*** | -8.0124*** | -7.2986*** |
|  | (3.1417) | (-8.0640) | (-7.0170) | (-14.9749) | (-13.7597) |
| *Control* | Yes | Yes | Yes | Yes | Yes |
| *Industry* | Yes | Yes | Yes | Yes | Yes |
| *Year* | Yes | Yes | Yes | Yes | Yes |
| N | 25605 | 19762 | 19762 | 29630 | 29630 |
| Adj R-sq | 0.1877 | 0.5353 | 0.5484 | 0.6070 | 0.6150 |

**6.1.2 Industry chain transmission effect**

The theoretical analysis in the previous section suggests that through industry chain transmission, labour hiring jobs of industrial technological advances can be transmitted to companies upstream and downstream of the industry chain. This paper identifies the relatively important downstream and upstream industries of each industry according to China's input-output table (2010) as follows: for industry i, if more than 1 per cent of the output of industry i is put into another industry k for use, and the proportion of the output of industry k that is put into industry i is not more than 1 per cent, industry k is considered to be a relatively important downstream industry of industry j, and industry j is a relatively important upstream industry of industry k.

|  |  | （3） |
| --- | --- | --- |

The tfp_ct_ coefficient γ^own^ reflects the impact of industrial technological advances on the labour employment of companies in industry i. The Upstream_ct_ coefficient γ^up^ measures the impact of industrial technological advances on the labour employment of companies in the downstream industries of industry i, which is known as the ‘upstream transmission effect’, while the Downstream_ct_ coefficient γ^down^ reflects the impact of industrial technological advances in the upstream industries of industry i on the labour demand of companies in industry i, which is known as the ‘downstream transmission effect’. The coefficient γup of Downstreamct reflects the impact of industrial technological advances in the upstream industries of industry i on the labour demand of companies in industry i, which is called the ‘downstream transmission effect’. In this paper, drawing on Acemoglu et al. (2016), Upstream_ct_ and Downstream_ct_ are constructed as follows:

|  | $U\text{p}stream_{ct}=\text{tfp}_{\text{ct}}*\sum_{\text{k}} (output\%_{i\to k}^{2010})$ | （4） |
| --- | --- | --- |
|  | $\text{Down}stream_{ct}=\text{tfp}_{\text{ct}}*\sum_{\text{k}} (\text{in}put\%_{i\to k}^{2010})$ | （5） |

Where $\sum_{\text{k}} (output\%_{i\to k}^{2010})$ denotes the correlation coefficient between industry i and its downstream industry k, reflecting the share of industry i's use of inputs to industry k per unit of output, and $\sum_{\text{k}} (\text{in}put\%_{i\to k}^{2010})$ is the correlation coefficient between industry i and its upstream industry k, reflecting the share of intermediate products of industry k per unit of industry i's output.

Columns (3) and (5) of Table 9 control for industrial technological advances in this industry. The results show that the coefficients of industrial technological advances on the labour demand of companies in both upstream and downstream industries are significantly negative, indicating the existence of both upstream and downstream transmission effects.

**6.2 Inhibitory Effects: A Test of Resource Taking Effect and Hiring Delay Effect**

**6.2.1 Resource curse effect**

The previous theoretical analysis that industrial technology advancement produces investment expansion effect and inhibits company labour employment. Enterprises that take the initiative to upgrade industrial technology are bound to increase equipment investment, talent investment and R & D investment during the transition period, for this reason, this paper adopts the enterprise free cash flow measure of enterprise capital capacity, Table 10, column (1) columns of the regression of industrial technological advances on the capital capacity of the enterprise, the results show that industrial technological advances on the coefficient of free cash flow of the enterprise at the level of 5 per cent significantly negative, which indicates that there is an effect of capital hogging.

Table10 Tests for resource usage effects and hiring delay effects

|  | （1）  Free cash flow of the company | （2）  *F.lnNumber* | （3）  *F2.lnNumber* |
| --- | --- | --- | --- |
| *tfp* | -30.0091** | -0.6100*** | -0.5682*** |
|  | (-2.3105) | (-22.4067) | (-19.3182) |
| *_cons* | -329.4713 | -7.0287*** | -7.1059*** |
|  | (-1.4776) | (-14.5766) | (-12.8130) |
| *Control* | Yes | Yes | Yes |
| Industry | Yes | Yes | Yes |
| Year | Yes | Yes | Yes |
| N | 40571 | 35958 | 31689 |
| Adj R-sq | 0.0126 | 0.5917 | 0.5670 |

**6.2.2 Delayed employment effect**

The previous theoretical analysis suggests that the impact of corporate technological advances on companies may have a certain employment delay effect, the explanatory variables are used in the future period and the next two periods of corporate labour employment, the results of Table 10 Columns (2), Columns (3) show that the tfp coefficients are significantly negative at the level of 1%, which suggests that the industrial technological advances significantly inhibit the employment of company labour in the future period and in the next two periods of time, and that there is a hiring delay effect.

**6. There are also some writing errors in this article, for example, double is not required in front of difference-in-difference model.**

**Response：**Thank you for pointing out the writing errors, which have been corrected by the author.

**Reviewer #2**

1. **The title should be more precise and represent the specifics of your study. Your study concentrates on Chinese industry, and hence, this should be in the title as well, e.g., "Technological Advance in Industry and the Demand for Labour Employment in China"**

**Response：**As you suggested, the title have be changed to ‘Industrial Technological Advance and the Employment Demand for China's Labour Force—Micro Evidence from Labour Hiring in Companies’.

2. **The general development of technology is known as "Industry 4.0" or the fourth industrial revolution. This should be elaborated and analyzed in the literature review. Also, some papers are already dealing with the upcoming Industry 5.0, and I suggest you consider browsing literature on this topic, too.**

**Response：**Thanks to your suggestion, this paper further references literature on robotics applications, artificial intelligence, automation, and digital transformation.

3. **Throughout the entire paper, you emphasize the general term "industry". Can you prove that technological development in all industries in China is: Reported? Has the same effects that you hypothesized in H1a and H1b?**

**Response：**H1a and H1b are competing hypotheses. In the field of empirical analysis, we often think about the accuracy and completeness of existing hypotheses, and competing hypothesis testing assists us in selecting the most probable explanation by comparing multiple possible explanations and emphasising the relative likelihood between the hypotheses, thus providing new ideas for strengthening causation and refining the analysis of mechanisms. For example, the article *CEO Overconfidence and Bonus Target Ratcheting* published by The Accounting Review on 17 May 2024 uses competing hypothesis testing with the following specific hypotheses:

H2a: The degree of asymmetric target ratcheting is greater for overconfident CEOs than for non-overconfident CEOs.

H2b: The degree of asymmetric target ratcheting is smaller for overconfident CEOs than for non-overconfident CEOs.

4. **You have a biased population sampling by selecting A-list companies from two rather greatly populated and developed cities. In statistics, this is considered a doubtful specimen and requires a thorough explanation of why this sample is representative.**

**Response：**There are two considerations for selecting A-share listed companies in Shanghai and Shenzhen: firstly, listed companies are the outstanding representatives of selected Chinese companies, which are the ‘basic discs’ of the national real economy and the main promoters of technological advancement. Listed companies are not only the key driving force for China's high-quality economic development, but also an important component of the national innovation system and an important source of industrial science and technology innovation. Secondly, the data of listed companies are publicly available, with a high degree of information disclosure, which can well test our theoretical viewpoints.

5. **In equations (1) and (2), index i is not defined. Mathematically, it should be written as lnNumber(t) if it is in function for a certain year.**

**Response：**Thank you for your valuable comments. lnNumber_it_ is the labour employment of company i in year t. The authors have explicitly indexed the definition of i in the text, with i denoting an individual company.

6. **You refer to tables with data in the text. However, there are no tables in the attachment or the text.**

**Response：**Following your suggestions, the authors have included tables in the text, listed after the first cited paragraph, added the title of the supporting information document at the end of the manuscript, and updated the text citation accordingly.

**Reviewer #3**

**1.methodologically, the article is correct. The estimation method used and the robustness check tools are correct. And the results obtained prove the reliability of the study. The conclusions drawn from the numerical analysis are also valid.**

**Response：**Thank you for acknowledging the article.
